# The Impact of Minimal Sunlight Exposure on Bone Health: Insights From a Cohort Study in Erythropoietic Protoporphyria

**DOI:** 10.1210/clinem/dgae729

**Published:** 2024-10-14

**Authors:** Louisa G Kluijver, Margreet A E M Wagenmakers, J H Paul Wilson, Janneke G Langendonk

**Affiliations:** Porphyria Center Rotterdam, Center for Lysosomal and Metabolic Disease, Department of Internal Medicine, Erasmus MC, Erasmus University Medical Center, 3000 WB, Rotterdam, The Netherlands; Porphyria Center Rotterdam, Center for Lysosomal and Metabolic Disease, Department of Internal Medicine, Erasmus MC, Erasmus University Medical Center, 3000 WB, Rotterdam, The Netherlands; Porphyria Center Rotterdam, Center for Lysosomal and Metabolic Disease, Department of Internal Medicine, Erasmus MC, Erasmus University Medical Center, 3000 WB, Rotterdam, The Netherlands; Porphyria Center Rotterdam, Center for Lysosomal and Metabolic Disease, Department of Internal Medicine, Erasmus MC, Erasmus University Medical Center, 3000 WB, Rotterdam, The Netherlands

**Keywords:** erythropoietic protoporphyria, osteoporosis, vitamin D, cholecalciferol, bone mineral density, sunlight

## Abstract

**Context:**

Erythropoietic protoporphyria (EPP) is a rare inherited metabolic disease, causing lifelong painful phototoxic reactions, minimal sunlight exposure, and vitamin D deficiency. Previous studies reported a high osteoporosis prevalence in EPP patients.

**Objective:**

To identify those at risk for low bone mineral density (BMD) and assess which factors, including treatment with cholecalciferol and afamelanotide, improve BMD in EPP.

**Methods:**

A longitudinal ambispective single-center cohort study. Data from patient files and two-time questionnaires from adult patients with EPP who underwent at least one dual-energy x-ray absorptiometry (DXA) scan between 2012 and 2023 were used.

**Results:**

BMD is low in EPP patients, with 82.7% of the 139 patients having a Z-score below 0 SD at baseline. Low BMD classified as osteopenia was found in 39.5%, and osteoporosis in 15.3%. There were 50 osteoporosis-related fractures in 34.2% of patients. Aging (odds ratio [OR] 1.08; CI, 1.03-1.12), persistent vitamin D deficiency (OR 1.11; 95% CI, 1.00-1.23) and a low body mass index (OR 0.91; 95% CI, 0.82-0.99) increased the odds of low BMD. Patients with a vitamin D deficiency (OR 5.51; 95% CI, 1.69-17.92) and no cholecalciferol at baseline (OR 0.22; 95% CI, 0.04-1.34) had the highest odds of improving their BMD. Afamelanotide did not improve BMD.

**Conclusion:**

25-hydroxyvitamin D (25(OH)D) status plays a crucial role in both preventing low BMD and improving BMD. EPP is a natural model for lack of sunlight exposure and vitamin D deficiency, underlining the importance of lifelong adequate vitamin D status for bone health in the general population.

Erythropoietic protoporphyria (EPP) is a rare inherited metabolic disorder of heme biosynthesis, leading to lifelong painful phototoxic reactions starting in childhood. Patients rely on sun-protective clothing and sun-avoiding behavior to avoid these reactions. The lack of sunlight exposure, as seen in the EPP population, increases the risk of vitamin D deficiency (1), a known risk factor for early-onset osteoporosis (2). Previous small cross-sectional studies among EPP patients showed a high prevalence of vitamin D deficiency (3-6), as well as low bone mineral density (BMD) classified as osteopenia (36%) and osteoporosis (23%) based on dual-energy x-ray absorptiometry (DXA) scan T-scores (6). At the time of these studies, no treatment to ameliorate symptoms of EPP was available, and cholecalciferol (vitamin D3) prescription was not standard of care.

Since 2016, afamelanotide, a potent α-melanocyte-stimulating hormone (α-MSH) synthetic analogue that increases the production of eumelanin by agonistically binding to the melanocortin-1 receptor (MC1R), has been available for the treatment of adult patients with EPP (7). Afamelanotide mitigates the phototoxic symptoms caused by EPP and has been shown to increase time spent outdoors because patients can endure more light exposure (8, 9). Currently no longitudinal data on BMD in EPP patients are available. Furthermore, insights into which specific factors increase the risk of low BMD in EPP patients or how treatment with afamelanotide and cholecalciferol impacts their BMD are lacking. In a previously published study, we found that cholecalciferol supplementation increases 25-hydroxyvitamin D (25(OH)D) levels in patients with EPP, while treatment with afamelanotide does not. However, this study did not investigate the effect of both treatments on BMD.

Moreover, there is ongoing controversy regarding the efficacy of cholecalciferol supplementation for improving BMD in the general population (10). Recent meta-analysis of all intervention studies in relatively healthy adults demonstrated that cholecalciferol significantly increases BMD when the data from all studies were pooled (11-13). However, no significant improvement was observed in individuals under 65 years of age, and multiple studies reported no positive effect on BMD. Given that patients with EPP are otherwise healthy, they are an ideal cohort to investigate the effects of cholecalciferol supplementation on BMD.

The primary objective of this study is to identify adult EPP patients who are at greater risk of developing low BMD and assess what factors, including treatment with cholecalciferol and afamelanotide, are associated with the improvement of their BMD. We hypothesize that patients who are severely affected by their EPP, resulting in minimal sunlight exposure and ongoing vitamin D deficiency, are most at risk for low BMD. Furthermore, we expect that cholecalciferol and bisphosphonate treatment will improve BMD. Lastly, it is plausible that afamelanotide treatment can increase physical activity and 25(OH)D levels due to increased sun exposure, which could lead to improvement in BMD.

## Methods

### Study Design, Setting, and Participants

In this ambispective longitudinal single-center cohort study, all adult patients with EPP who visited the Erasmus Medical Centre (Erasmus MC) in Rotterdam, the Netherlands, and underwent at least one DXA scan were eligible for inclusion. The Erasmus MC is the leading expert center in the Netherlands for porphyria, providing follow-up and care for all Dutch EPP patients. Included were patients aged 16 years and older, with a confirmed diagnosis of EPP based on phototoxic symptoms and increased erythrocyte protoporphyrin IX levels (> 4 times upper limit of normal). Ethics approval was granted by the Ethics Committee of Erasmus Medical Centre (MEC 2011-525).

### Data Collection

From 2014 until 2022, patients prospectively filled in questionnaires, before receiving treatment with afamelanotide. Follow-up questionnaires were collected in 2022 and 2023, when most patients were receiving afamelanotide. Patients reported on smoking behavior, alcohol consumption, comorbidities, glucocorticoid use, history of fractures, and parental hip fracture occurrence. The questionnaires included bone-specific physical activity questionnaire (BPAQ) to calculate the current, past, and total bone-specific physical activity scores (6, 14, 15).

Patient data were collected retrospectively from medical records and included date of birth, sex, weight, height, BMD, T- and Z-scores from the lumbar spine and femoral neck, and serum 25(OH)D measurements. If no 25(OH)D measurements were available at the time of the DXA scan, measurements from the previous 6 months were used. Prescription dates for vitamin D3 supplements (cholecalciferol), osteoporosis treatment (bisphosphonates, denosumab or teriparatide), and afamelanotide were also retrieved. Fracture history details, including site, cause, and year of occurrence, were collected. Records were examined for secondary causes of osteoporosis, such as glucocorticoid use, hyperthyroidism, hyperparathyroidism, Cushing syndrome, and hypogonadism.

### Routine Care

As part of routine care, reflecting real-world clinical practice, all patients underwent a DXA scan. Follow-up DXA scans were done every 2 to 5 years in patients with T-scores classifying as osteopenia or osteoporosis or were done based on individual indications. All DXA scans in this study were performed between August 2012 and July 2023. The majority of EPP patients started with afamelanotide treatment in 2016, leading to hospital visits 1 to 4 times annually for treatment administration. Laboratory measurements, including 25(OH)D levels, were obtained at least once a year. If vitamin D deficiency (25(OH)D < 50 nmol/L, < 20 ng/mL) was present, cholecalciferol was prescribed and patients received counseling from their treating physician. All patients received either an 800 to 1000 international units (IU) daily dose or a 50 000 IU monthly dose of cholecalciferol.

### Outcome Measures

The main outcome was BMD reported as T- and Z-scores, assessed at the femoral neck and lumbar spine. T-scores were based on National Health and Nutrition Examination Survey and Lunar reference populations aged 20 to 30 and 20 to 40 years, respectively. Z-scores were adjusted for patients age, sex, and ethnicity. Following the World Health Organization (WHO) osteoporosis guidelines, results were categorized using T-scores: normal BMD (T-score ≥ −1 SD), osteopenia (T-score between −1 and −2.5 SD), or osteoporosis (T-score ≤ −2.5 SD) (16). For young adults aged 20 years and below, the Z-scores below −2 SD were categorized as osteoporosis, and those below −1 SD were considered the lower range of normal, categorized as osteopenia (17).

During follow-up DXA scans, changes in BMD were assessed as percentages compared to baseline. Based on national guidelines, these percentages were then categorized as unchanged, increased, or decreased BMD (18, 19). Changes were deemed significant when they exceeded the minimum threshold of significant change, known as the *Least Significance Change*. For the femoral neck, this was 4% to 5% and for the lumbar spine 5% to 6% (18, 19). Smaller changes were categorized as unchanged.

A combined conclusion from both sites, the femoral neck and lumbar spine, was created as the main outcome. If either site had osteopenia or osteoporosis, a patient was categorized as such. Only if both sites had a normal BMD, it was categorized as “normal.” If either site had an increase or decrease in BMD at follow-up, it was categorized as such (“increased,” “decreased”) or both (“increased/decreased”). If BMD at both sites did not change compared to baseline, it was categorized as “unchanged.”

### Fracture Classification

Fractures were classified into 2 categories based on their site (20). Osteoporosis-related fractures include vertebral, hip, wrist-forearm, humeral, rib, pelvic, femoral, tibia, fibula, clavicle, scapula, and sternum fractures. All other fractures including skull, hand, finger, foot, toe, ankle, and patella fractures were considered unrelated to osteoporosis.

### Predictors and Risk Factors

Patient age at time of DXA scan and time between DXA scans were calculated using dates. Smoking was categorized as never smoking, past smoking, or current smoking. Alcohol consumption classification followed guidelines from the alcohol expert center in the Netherlands, defining excessive consumption as ≥ 14 units/week for women or ≥ 21 units/week for men (21). Disease severity was based on “time to symptoms,” the self-reported time in direct sunlight before experiencing the first phototoxic symptoms prior to afamelanotide treatment. The variable was categorized based on the median, with “severe disease” defined as a time to symptoms < 15 minutes and “mild disease” defined as a time to symptoms ≥ 15 minutes.

Vitamin D deficiency was defined as 25(OH)D < 50 nmol/L (< 20 ng/mL) according to the clinical practice guidelines of the Endocrine Society Taskforce (22). Severe vitamin D deficiency was defined as < 30 nmol/L (< 12 ng/mL), associated with an increased risk of osteomalacia and nutritional rickets (1, 23). The vitamin D deficiency score was computed as the percentage of 25(OH)D measurements that were below the threshold of < 50 nmol/L up to the day of the DXA scan.

Variables were created indicating whether the patients had initiated cholecalciferol, bisphosphonate, or afamelanotide treatment at time of DXA scan. Patients were categorized as on treatment (cholecalciferol or afamelanotide) at baseline if the first prescription was issued before the baseline DXA scan, otherwise, they were categorized as not on treatment.

### FRAX and BPAQ Assessment

Fracture risk scores were calculated using the fracture risk assessment tool (FRAX) (24). The WHO-developed FRAX tool, was utilized to calculate the 10-year probability of experiencing either a hip or other major osteoporotic fractures, including vertebral, radius, and humeral fractures. This scoring system was based on patient characteristics, risk factors, and femoral neck BMD. FRAX scores are considered elevated when the risk estimate is ≥ 3% for the hip and/or a risk of ≥ 20% for other major osteoporotic fractures (24).

The BPAQ score estimated bone strength in clinically significant areas based on specific types, frequency, and age at time of physical activity. Three scores were derived: the current, past, and total BPAQ, where the current score covered activities over the last 12 months, the past score covered all physical activity from childhood onward, and the total score considered both. In healthy reference populations, BPAQ scores positively correlated with BMD (6, 14, 15).

### Data Management and Validation

Data were entered and stored in the database program Castor version 2022.5.2.0 from 2021 to 2023. The 25(OH)D measurements were exported from electronic patient files and imported directly into Castor. Database validation check was performed in 10% of all data chosen at random by a fellow investigator; an error percentage of < 5% was considered acceptable.

Data on 25(OH)D measurements previously published in a multicenter cohort study (25), included this Dutch population as well as another cohort. While there is some overlap, the objectives and analytic approaches differ. In the previous study we observed that cholecalciferol increases 25(OH)D levels, while treatment with afamelanotide did not. The current study aims to investigate the effect of both treatments on BMD.

### Statistical Analysis

For descriptive analyses, data were summarized using mean and SD for parametric data, median and interquartile range for nonparametric data, and frequencies and percentages for categorical data. Depending on the distribution, Student *t* test or Wilcoxon Signed Rank test was used to compare 2 groups. ANOVA or Kruskall-Wallis test was used to compare more than 2 groups. Chi-squared test was used when comparing categorical data. Correlations were assessed using the Spearman correlation test. Statistical tests with *P* values were 2-sided with a significance level of .05.

We used 2 multinomial logistic regression models to examine the association between the predictors and the outcome. The baseline model aimed to identify factors associated with the risk for osteoporosis, categorizing outcomes into; normal BMD, osteopenia, and osteoporosis. Predictors included age, sex, BMI, disease severity, and vitamin D deficiency score. The follow-up model identified factors associated with changes in BMD categorized as; unchanged, increased, or decreased BMD. Predictors for this model included: sex, BMI, baseline vitamin D deficiency, and treatments with cholecalciferol and afamelanotide at baseline. The reference category for the analysis was normal for the first model and unchanged BMD for the second model. Cases with missing data on predictors and outcome were excluded from analysis. Details on predictor selection can be found in the supplements.

The follow-up model and correlation analysis excluded cases with a prior or current bisphosphonate or denosumab use to evaluate the true effect of cholecalciferol and afamelanotide. Furthermore, patients that had an increase at one site and decrease at the other were excluded from the follow-up model analysis. Patients with sufficient 25(OH)D levels at baseline were excluded from correlation analysis.

All statistical analysis were performed using RStudio, version 2021.09.2. The “mlogit” package was utilized to estimate the multinomial logistic regression model (26).

## Results

**Figure 1.**
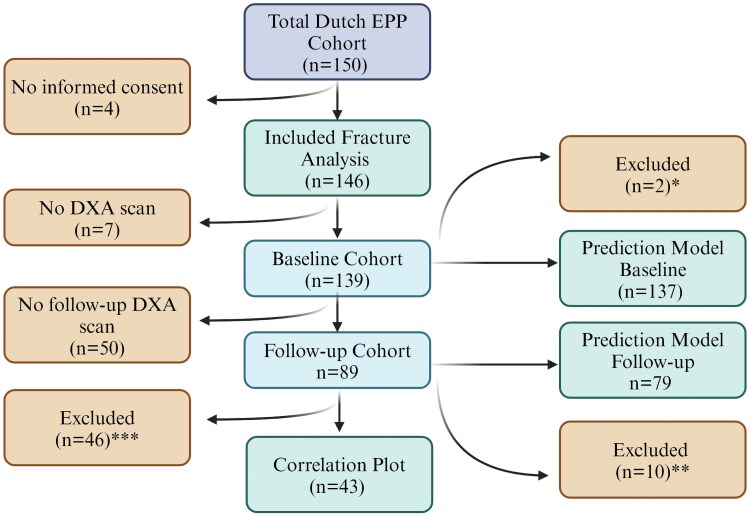
Flowchart. *Two patients were excluded from the baseline model predicting osteoporosis and osteopenia due to missing values on vitamin D deficiency score. **Ten patients were excluded from the follow-up model predicting increase or decrease in BMD due to various reasons, including both an increase and decrease of BMD at different sites, missing values on vitamin D status and 7 patients using bisphosphonates. This second model excluded cases with a history of prior bisphosphonate use or those currently using bisphosphonates, to evaluate the true effect of cholecalciferol and afamelanotide. There were no vertebral compression fractures that could explain this increase in BMD at the lumbar spine, there was 1 patient's report mentioning potential overestimation due to degenerative abnormalities. ***A total of 46 patients were excluded for the correlation plot analysis between the delta z-sores and vitamin D deficiency score for various reasons. Those with a normal vitamin D status at baseline were excluded (n = 33), those using bisphosphonates or other treatment for osteoporosis were excluded (n = 9), and lastly missing value for the vitamin D deficiency score was excluded (n = 1). Created with BioRender.com.

We included 146 patients to assess fracture prevalence (97.3% Dutch EPP cohort), only excluding those without informed consent. The BMD analysis excluded patients without a DXA scan, resulting in a baseline sample of 139 patients (92.6%) and 89 patients (59.3%) with a follow-up DXA scan (Fig. 1).

Patient characteristics at baseline and follow-up are summarized in Table 1. At baseline, the mean age was 39.5 (15.2) years, with 36 (25.9%) patients aged over 50. Females constituted half of the population (n = 69 [49.6%]), with 19 patients (13.7%) aged over 50. The mean BMI was 24.8 (4.4) kg/m^2^, with 3 patients (2.2%) categorized as underweight, 41 (29.5%) as overweight, and 20 (14.4%) as obese.

**Table 1.** Patient Characteristics

	Baseline DXA scan (n = 139)	Follow-up DXA scan (n = 89)
Time to follow-up, months	..	42.4 (19.8)
Range, months	..	12-85
Age, years	39.5 (15.2)	46.4 (15.2)
< 50 years	103 (74.1%)	59 (66.3%)
≥ 50 years	36 (25.9%)	30 (33.7%)
Sex, male	70 (50.4%)	46 (51.7%)
Body mass index, kg/m^2^	24.8 (4.4)	24.9 (4.3)
Underweight (< 18.5)	3 (2.2%)	0 (0.0%
Normal (≥ 18.5-24.9)	75 (54.0%)	49 (55.1%
Overweight (≥ 25.0-29.9)	41 (29.5%)	28 (31.5%
Obese (≥ 30)	20 (14.4%)	12 (13.5%
Smoking*^a^* *missing*	*23* (*16.5%)*	*8* (*9.0%)*
Never	62 (44.6%)	40 (44.9%)
Current	30 (21.6%)	22 (24.7%)
Passed	24 (17.3%)	19 (21.3%)
Excessive alcohol use*^b^* *missing*	4 (2.9%) *54 (38.8%)*	3 (3.4%) *29 (32.6%)*
Rheumatic disease*^a,c^* *missing*	3 (2.2%) *26 (18.7%)*	2 (2.2% *11 (12.4%*
Corticosteroid use*^a^*	13 (9.4%)	10 (11.2%)
Oral long-term	2 (1.4%)	2 (2.3%)
Oral short-term	1 (0.7%)	1 (1.1%)
Cutaneous	4 (2.9%)	3 (3.4%)
Aerosol, nose-spray	2 (1.4%)	1 (1.1%)
Unknown*^d^*	4 (2.9%)	3 (3.4)%
Diabetes mellitus*^a^* *missing*	2 (1.4%) *26 (18.7%)*	3 (3.3%) *10 (11.2%)*
Hip fracture parents*^a^* *missing*	4 (2.9%) *25 (18.0%)*	3 (3.4%) *10 (11.2%)*
BPAQ*^a^* *missing*	*23* (*16.5%)*	*7* (*7.9%)*
Total BPAQ*^e^*	10.8 (3.8-22.2)	9.6 (1.8-21.6)
Past BPAQ*^e^*	19.6 (6.4-41.4)	18.2 (2.0-43.2)
Current BPAQ*^e^*	0.7 (0.2-3.3)	0.7 (0.3-2.9)
Physical activity, times per week*^a,e^* *missing*	3.0 (1.0-6.0) *24 (17.3%)*	3.0 (2.0-6.0) *8 (9.0%)*
Time to symptoms, minutes*^a,e^* *missing*	15.0 (8.5-30.0) *1 (0.7%)*	15.0 (7.0-30.0) *0 (0.0%)*
Mild, > 15 minutes	72 (51.8%)	46 (51.7%
Severe, ≤ 15 minutes	66 (47.5%)	43 (48.3%
Baseline 25(OH)vitamin D*^e^* *missing*	43.0 (30.0-60.0) *2 (1.4%)*	40.0 (26.8-60.0) *1 (1.1%)*
Normal (≥ 50 nmol/L)	55 (39.6%)	35 (71.9%)
Deficiency (< 50 nmol/L)	82 (59.0%	53 (27.0%)
Cholecalciferol prescription	41 (29.5%)	76 (85.4%)
Afamelanotide prescription	34 (24.5%)	79 (88.8%)
Bisphosphonate prescription		
Current	0 (0.0%)	5 (5.6%)
Past	1 (0.7%)	3 (3.4%)

All data are reported in number (percentages) for categorical values or mean (SD) for continuous variables unless otherwise specified.

^*a*^Self-reported.

^*b*^Self-reported, excessive alcohol use was defined as ≥14 units per week for women and ≥21 unites per week for men.

^*c*^Polymyalgia rheumatica (PMR), rheumatoid arthritis, ankylosing spondylitis.

^*d*^Unknown route of administration.

^*e*^Median (interquartile range).

Abbreviations: .., not applicable; BMI, body mass index; BPAQ, bone-specific physical activity questionnaire; DXA, dual-energy x-ray absorptiometry.

**Figure 2.**
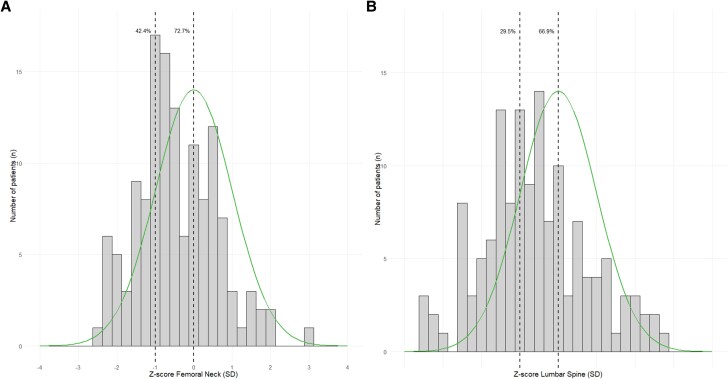
Distribution of DXA scan Z-scores at baseline. These figures show the distribution of the baseline Z-scores of the EPP population. The solid line illustrates the standard normal distribution of the healthy population of the same age, sex, and ethnicity. The dashed lines are placed at 0 SD and −1 SD respectively. (A) the Z-scores of the femoral neck are displayed, showing that 72.7% falls below 0 SD and 42.4% falls below −1 SD. (B) The Z-scores of the lumbar spine are displayed, illustrating that 66.9% is below 0 SD and 29.5% is below −1 SD.

The prevalence of low BMD, classified as osteopenia based on T-scores, was 39.5%, while osteoporosis affected 15.3% of patients (Table 2). A Z-score below 0 SD was observed in 82.7% patients and 48.2% below −1 SD (Table 2, Fig. 2). At follow-up, 24.7% showed an increase in BMD, 26.9% a decrease, and 2.2% showed an increase at one site and decrease at the other. The delta Z-scores increased for both the femoral neck (median 0.1, interquartile range [IQR]:-0.2-0.1) and lumbar spine (median 0.2, IQR: −0.4-0.3).

**Table 2.** Prevalence of osteoporosis in erythropoietic protoporphyria and changes in bone mineral density at follow-up

	Baseline DXA scan (n = 139)	Follow-up DXA scan (n = 89)
Conclusion DXA scan		
Normal	63 (45.3%)	30 (33.7%)
Osteopenia	55 (39.6%)	43 (48.3%)
Osteoporosis	21 (15.1%)	16 (18.0%)
Z-score femoral neck *missing*	−0.4 (1.0) *5 (3.6%)*	−0.5 (1.1) *1 (1.1%)*
Below 0 SD	94 (67.6%)	60 (67.4%)
Below −1 SD	42 (30.2%)	31 (34.8%)
Z-score lumbar spine	−0.7 (1.4)	−0.7 (1.7)
Below 0 SD	101 (72.7%)	61 (68.5%)
Below −1 SD	59 (42.4%)	47 (52.8%)
Z-score at either site		
Below 0 SD	115 (82.7%)	71 (79.8%)
Below −1 SD	67 (48.2%)	52 (58.4%)
Delta Z-score*^a^*	..	
Femoral neck	..	0.1 (−0.2 to 0.1)
Lumbar spine	..	0.2 (−0.4 to 0.3)
BMD changes at either site*^b^*	..	
Unchanged	..	41 (46.1%)
Increase	..	22 (24.7%)
Decrease	..	24 (27.0%)
Increase/Decrease*^c^*	..	2 (2.2%)

All data are reported in number (percentages) for categorical values or mean (SD) for continuous variables unless otherwise specified.

^*a*^Median (interquartile range).

^*b*^BMD changes compared to baseline, unchanged, significant increase or decrease as specified in methods.

^*c*^Increase at one site and decrease at the other.

Abbreviations: .., not applicable; BMD, bone mineral density; DXA, dual-energy x-ray absorptiometry.

Among those aged ≥ 50 years, 33.3% had osteoporosis (Table 3). The youngest patient with osteopenia was 16 years old, whereas the youngest with osteoporosis was 24 years old. Cases with osteoporosis had a significantly higher mean age of 51.2 (13.8) years, compared to 37.4 (14.3) years in those with a normal BMD (*P* < 0.01). In the osteoporosis group, gender distribution was equal between men and women 52.4% vs 47.6%.

**Table 3.** Patient characteristics categorized based on the conclusion of the baseline DXA scan

	Normal BMD	Osteopenia	Osteoporosis	Overall	*P* value
Patients, n (%)	63 (45.3%)	55 (39.5%)	21 (15.3%)	139 (100%)	
Age, years	37.4 (14.3)	37.3 (15.0)	51.2 (13.8)	39.5 (15.2)	<.01*
< 18 years	4 (6.3%)	1 (1.8%)	0 (0.0%)	5 (3.6%)	
20-40 years	21 (33.3%)	29 (52.7%)	6 (28.6%)	56 (40.3%)	
< 50 years	52 (82.5%)	42 (76.4%)	9 (42.9%)	103 (74.1%)	
≥ 50 years	11 (17.5%)	13 (23.6%)	12 (57.1%)	36 (25.9%)	
Range, years	16-70	16-70	24-71	16-71	
Sex, male	34 (54.0%)	26 (47.3%)	11 (52.4%)	71 (51.1%)	.76
Menopause*^b^*	5 (7.9%)	7 (12.7%)	7 (33.3%)	19 (13.7%)	.01*
BMI, kg/m^2^	25.5 (4.5)	24.0 (4.57)	25.1(3.5)	24.8(4.4)	.16
Underweight (< 18.5)	1 (1.6%)	2 (3.6%)	0 (0.0%)	3 (2.2%)	.19
Normal (≥ 18.5-24.9)	31 (49.2%)	34 (61.8%)	10 (47.6%)	75 (54.0%)	
Overweight (≥ 25.0-29.9)	18 (28.6%)	13 (23.6%)	10 (47.6%)	41 (29.5%)	
Obese (≥ 30)	13 (20.6%)	6 (10.9%)	1 (4.8%)	20 (14.4%)	
Smoking*^a^* *missing*	*13* (*20.6%)*	*6* (*10.9%)*	*4* (*19.0%)*	*23* (*16.5%)*	.82
Never	27 (42.9%)	26 (47.3%)	9 (42.9%)	62 (44.6%)	
Current	11 (17.5%)	15 (27.3%)	4 (19.0%)	30 (21.6%)	
Former	12 (19.0%)	8 (14.5%)	4(19.0%)	24 (17.3%)	
Excessive alcohol use*^c^* *missing*	2 (3.2%) *24 (38.1%)*	0 (0.0%) *19 (34.5%)*	2 (9.5%) *11 (52.4%)*	4 (2.9%) *54 (38.8%)*	.03*
Diabetes mellitus*^a^* *missing*	3(4.8%) *15 (23.8%)*	0 (0.0%) *7 (12.7%)*	0 (0.0%) *4 (19.0%)*	3 (2.2%) *26 (18.7%)*	
Rheumatic disease*^a,d^* *missing*	2 (3.0%) *15 (23.8%)*	1 (1.8%) *7 (12.7%)*	0 (0.0%) *4 (19.0%)*	3 (2.2%) *26 (18.7%)*	
Hypogonadism*^e^*	1 (1.6%)	0 (0.0%)	0 (0.0%)	1 (0.7%)	
Hyperparathyroidism*^f^*	1 (1.6%)	0 (0.0%)	0 (0.0%)	1 (0.7%)	
Corticosteroid use*^a^*	8 (12.7%)	4 (7.3%)	1 (4.8%)	13 (9.4%)	
BPAQ*^a^* *missing*	*13* (*19.6%)*	*6* (*10.9%)*	*4* (*19.0%)*	*23* (*16.5%)*	
Current activity*^g^*	0.9 (0.6-5.4)	0.6 (0.0-2.7)	0.4 (0.0-1.4)	0.7 (0.2-3.3)	.02*
Past activity*^g^*	24.6 (7.4-48.7)	17.3 (5.6-37.8)	19.0 (3.9-18.8)	19.6 (6.4-41.4)	.37
Total activity*^g^*	15.9 (4.0-27.3)	9.1 (3.7-21.8)	9.5 (2.0-14.9)	10.8 (3.8-22.2)	.24
Time to symptoms, minutes*^a,g^* *missing*	17.5 (10.0-30.0) *1 (1.6%)*	15.0 (6.0-37.5) *0 (0.0%)*	15.0 (8.0-30.0) *0 (0.0%)*	15.0 (8.5-30.0) *1 (0.7%)*	.83
Mild, ≥ 15 minutes	31 (49.2%)	30 (54.4%)	11 (52.4%)	72 (51.8%)	.88
Severe, < 15 minutes	31 (49.2%)	25 (45.5%)	10 (47.6%)	66 (47.5%)	
Baseline vitamin D*^h^* *missing*	47.0 (23.7) *1 (1.6%)*	44.3 (20.4) *1 (1.8%)*	48.9 (23.0) *0 (0.0%)*	46.2 (22.3) *2 (1.4%)*	.68
Normal (≥ 50 nmol/L)	25 (39.7%)	20 (36.4%)	10 (47.6%)	55 (39.6%)	.55
Deficiency (< 50 nmol/L)	26 (41.3%)	20 (36.4%)	5 (23.8%)	51 (36.7%)	
Severe deficiency (< 30 nmol/L)	11 (17.5%)	14 (25.5%)	6 (28.6%)	31 (22.3%)	
Vitamin D deficiency score*^g^*	0.6 (0.0-1.0)	1.0 (0.3-1.0)	0.5 (0.0-1.0)	0.7 (0.0-1.0)	.41
Cholecalciferol prescription	23 (36.5%)	13 (23.6%)	5 (23.8%)	41 (29.5%)	.26
Afamelanotide prescription	17 (27.0%)	12 (21.8%)	5 (23.8%)	34 (24.5%)	.80
Bisphosphonate prescription	0 (0.0%)	0 (0.0%)	1 (4.8%)	1 (0.7%)	.06

All data are reported in number (percentages) for categorical values or mean (SD) for continuous variables unless otherwise specified. Statistically significant *P* values are indicated with an asterisk (*).

^*a*^Self-reported.

^*b*^Females aged 50 years and older.

^*c*^Self-reported, excessive alcohol use was defined as ≥14 units per week for women and ≥21 unites per week for men.

^*d*^Polymyalgia rheumatic (PMR), rheumatoid arthritis, ankylosing spondylitis.

^e^Post chemotherapy related to breast cancer.

^f^Post bariatric surgery. *P* values were calculated with the ANOVA for parametric, Kruskall-Wallis for nonparametric, and chi-squared test for categorical values.

^g^Median (interquartile range).

^h^Serum 25-OH vitamin D measurement at time of baseline DXA scan.

Abbreviations: DXA, dual-energy x-ray absorptiometry; BMD, bone mineral density; BMI, body mass index; BPAQ, bone-specific physical activity questionnaire; FRAX; fracture risk assessment tool.

**Figure 3.**
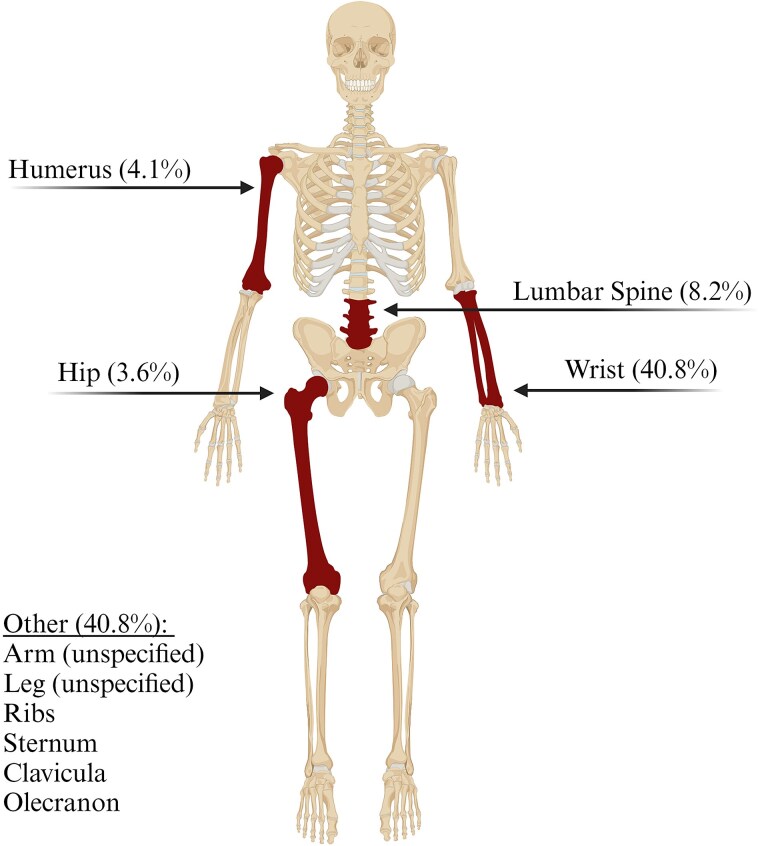
Overview of osteoporosis-related fractures sites in erythropoietic protoporphyria. Other: other osteoporosis-related fractures, excluding the 4 major osteoporotic fractures mentioned above. Created with BioRender.com.

Table 4 shows that 46.6% of patients reported a total of 99 fractures, with 34.2% patients experiencing fractures at sites associated with osteoporosis. 30.8% of the men, and 66.7% of the women aged ≥ 50 years had suffered an osteoporosis-related fracture (Table 5). The most commonly affected osteoporosis-related fracture site was the wrist (Fig. 3), with falls being the predominant cause.

**Table 4.** Prevalence of fractures based on osteoporosis-related sites in erythropoietic protoporphyria

	Non-osteoporosis-related fracture (n = 18)	Osteoporosis-related fracture*^a^* (n = 50)	Overall (n = 68)	*P* value
Prevalence in Dutch EPP cohort (%)	18 (12.3%)	50 (34.2%)	68 (46.6%)	
Age fracture, years *missing*	21.6 (12.0) *9 (50.0%)*	33.5 (21.2) *5 (10.0%)*	31.5 (20.3) *14 (20.6%*	.03
Sex, male	12 (66.7%)	22 (44.0%)	32 (47.1%	.17
Menopause*^b^* *missing*	0 (0.0%) *5 (27.8%)*	10 (20.0%) *0 (0.0%)*	10 (14.7% *5 (7.4%)*	.18
Conclusion DXA scan *missing*	*2* (*11.1%)*	*1* (*2.0%)*	*3* (*4.4%)*	.64
Normal	8 (44.4%)	18 (36.0%)	26 (38.2%)	
Osteopenia	5 (27.8%)	20 (40.0%)	25 (36.8%)	
Osteoporosis	3 (16.7%)	11 (22.0%)	14 (20.6%)	
Fracture site (%)				..
Hip	..	3 (6.0%)	3 (4.4%)	
Humeral	..	2 (4.0%)	2 (2.9%)	
Wrist	..	20 (40.0%)	20 (29.4%)	
Vertebral	..	5 (10.0%)	5 (7.4%)	
Other osteoporosis-related site*^c^*	..	10 (40.0%)	10 (29.4%)	
Toes, fingers, hands, feet	15 (83.3%)	..	15 (22.1%)	
Ankle, knee	2 (11.1%)	..	2 (2.9%)	
Skull	1 (5.6%)	..	1 (1.5%)	
Cause of fracture (%)				<.01*
Spontaneous	0 (0.0%)	1 (2.0%)	1 (1.5%)	.32
Bumping	2 (11.1%)	0 (0.0%)	2 (2.9%)	.16
Falling	3 (16.7%)	24 (48.0%)	27 (39.7%)	<.01*
Sports	5 (27.8%)	13 (26.0%)	18 (26.5%)	.06
Fighting	4 (22.2%)	0 (0.0%)	4 (5.9%)	.05*
Traffic accident	2 (11.1%)	5 (10.0%)	7 (10.3%)	.26
Other	2 (11.1%)	1 (2.0%)	3 (4.4%)	.56
Unknown	0 (0.0%)	6 (12.0%)	6 (8.8%)	.01*
Number of fractures (%)				.63
1	15 (83.3%)	35 (70.0%)	50 (73.5%)	
2	1 (5.6%)	9 (18.0%)	10 (14.7%)	
3	2 (11.1%)	4 (8.0%)	6 (8.8%)	
4	0 (0.0%)	1 (2.0%)	1 (1.5%)	
5	0 (0.0%)	1 (2.0%)	1 (1.5%)	
FRAX Score *missing*	*3* (*16.7%)*	*5* (*10.0%)*	*8* (*11.8%)*	
Hip ≥ 3%*^b^*	1 (5.6%)	2 (4.0%)	3 (4.4%)	1.00
FRAX score major osteoporotic fractures ≥ 20%*^b^*	0 (0.0%)	0 (0.0%)	0 (0.0%)	..

All data are reported in percentages for categorical values or mean (SD) for continuous variables unless otherwise specified.

*P* values were calculated with 2-sample *t* test for parametric and chi-squared test for categorical values. Statistically significant odds ratios are accompanied by their *P* values with an asterisk (*).

Abbreviations: .., not applicable; BMD, bone mineral density; DXA, dual-energy x-ray absorptiometry; EPP, erythropoietic protoporphyria; FRAX; fracture risk assessment score.

^*a*^Patients with fractures at both osteoporosis-related and not osteoporosis-related sites were classified as osteoporosis-related.

^*b*^Females aged 50 years and older. Threshold for increased fracture risk.

^*c*^Unspecified leg fracture (n = 5), ribs (n = 5), sternum (n = 1), coccyx (n = 1), clavicular (n = 2), fibula (n = 2), olecranon (n = 1).

**Table 5.** Prevalence of osteoporosis-related fractures in patients aged 50 years and older

	Men ≥ 50 years (n = 26)	Women ≥ 50 years (n = 30)	Overall ≥ 50 years (n = 56)	Healthy population
Age*^a^*	57.5 (54.0-64.5)	66.0 (56.5-69.0)	61.0 (55.0-67.5)	
Age fracture *missing*	19 (7.5-45.3) *16 (61.5%)*	50 (35.8-60.3) *10 (33.3%)*	46 (13.3-59.0) *26 (46.4%)*	
Osteoporosis-related fractures	8 (30.8%)	20 (66.7%)	23 (50.0%)	Men: 13%-22% Women: 40%-50% (20)
Fracture location				
Hip	0 (0.0%)	2 (6.7%)	2 (3.6%)	
Humerus	0 (0.0%)	0 (0.0%)	0 (0.0%)	
Wrist	4 (15.4%)	5 (16.7%)	9 (16.1%)	
Spine	1 (3.8%)	4 (13.3%)	5 (8.9%)	
Other osteoporosis-related	3 (11.5%)	9 (30.0%)	12 (21.4%)	
Bone mineral density (%) *missing*	*0* (*0.0%)*	*2* (*6.7%)*	*2* (*3.6%)*	
Normal	13 (50.0%)	10 (33.3%)	23 (41.1%)	
Osteopenia	7 (26.9%)	9 (30.0%)	16 (28.6%)	
Osteoporosis	6 (23.1%)	9 (30.0%)	16 (26.8%)	

All data are reported in number (percentages) for categorical values or median (interquartile range) for continuous values.

^*a*^Age at time of analysis (2023), to compare to the lifetime risk in the healthy population.

All EPP patients treated for osteoporosis, including 6 on bisphosphonates and 1 on denosumab, demonstrated a BMD increase, with 85.7% showing improvements at both sites. This proportion was significantly higher than the untreated group where only 14.2% increased (Tables 6 and 7).

**Table 6.** Effect of bisphosphonate and denosumab treatment on bone mineral density in patients with osteoporosis

	No treatment (n = 12)	Treatment*^a^* (n = 7)	*P* value
Age, years	45.5 (38.3-52.8)	66.0 (64.0-68.5)	
Changes in BMD*^b^*			< .01*^b^*
Unchanged	6 (42.9%)	0 (0.0%)	
Increase	1 (7.1%)	6 (85.7%)	
Increase/decrease	1 (7.1%)	1 (14.3%)	
Decrease	4 (28.6%)	0 (0.0%)	

All data are reported in number (percentages) for categorical values or median (interquartile range) for continuous variables. Two patients with osteoporosis were excluded due to not undergoing a follow-up DXA scan. Abbreviation: BMD, bone mineral density.

^*a*^Treatment included 6 patients with bisphosphonates and 1 with denosumab.

^*b*^Changes in BMD compared to baseline, the classification includes unchanged, significant increase, and/or a significant decrease, as specified in methods. The classification “Increase/Decrease” refers to increase at one site and decrease at the other. There were no vertebral compression fractures that could explain this increase at the lumbar spine; however, 1 patient’s report did mention potential overestimation by degenerative abnormalities.

*P* values were calculated with the chi-squared test for categorical values.

**Table 7.** Overview of patients with osteoporosis receiving bisphosphonate or denosumab Treatment

	Patient 1	Patient 2	Patient 3	Patient 4	Patient 5	Patient 6	Patient 7
Age	66	65	63	71	69	68	45
Sex	male	female	female	female	female	female	female
T-score femoral neck	−2.9	−2.0	−3.1	−2.4	−2.6	−2.4	−2.5
T-score lumbar spine	−2.7	−2.8	−4.8	−4.0	−2.3	−3.6	−3.4
Z-score femoral neck	−1.7	−0.5	−1.7	−0.9	−1.0	−0.8	−1.9
Z-score lumbar spine	−2.1	−1.2	−3.3	−2.3	−0.6	−1.9	−3.4
BMI	23.9	21.6	24.6	29.7	30.8	26.9	23.1
Fracture history	No	Yes	Yes	Yes	Yes	No	Yes
Fracture site	No	Pelvic fracture*^a^*	Hip	Spine	Hip	No	Ribs, coccyx*^b^*
Family history of hip fracture*^c^*	No	No	No	No	Unknown	No	No
Smoking	Current	Past	Never	Past	Unknown	Never	Never
Excessive alcohol	No	No	No	Unknown	Unknown	No	No
Medication/comorbidities	None	None	None	Prednisone	None	None	None
Risk factor Score*^d^*	2	2	2	4	2	1	1
Vitamin D baseline	26	81	91	63	19	71	65
Vitamin D deficiency score	1.00	0.00	0.33	0.00	1.00	0.00	0.00
Indication for bisphosphonates according to guidelines*^d^*	No	Yes	Yes	Yes	Yes	No	No
Osteoporosis treatment	Alendronic acid	Alendronic acid	Alendronic acid and denosumab	Alendronic acid and ibandronic acid	Alendronic acid	Alendronic acid	Alendronic acid

^*a*^Postoperational fracture for osteosarcoma.

^*b*^Trauma was fall from horse.

^*c*^Biological parent with hip fracture.

^*d*^Dutch guidelines osteoporosis and fracture prevention (27), this score includes the following risk factors: corticosteroid use, diabetes, rheumatoid arthritis, hypogonadism, hyperthyroidism, hyperparathyroidism.

**Figure 4.**
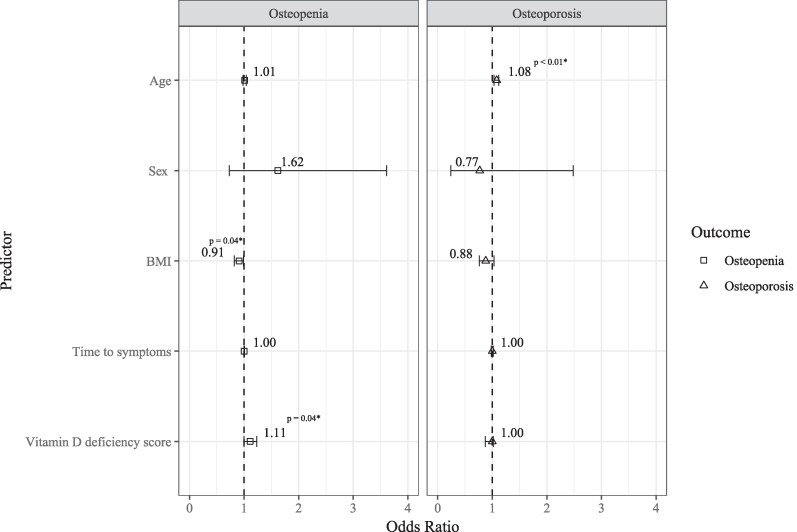
Odds ratios (OR) for predictors of osteopenia and osteoporosis risk. The figure displays the OR along with their corresponding CI, illustrating the associations between the predictors and 2 outcomes of osteopenia and osteoporosis, compared to the reference category normal bone mineral density. The squares and triangles depict the odds ratios for osteopenia and osteoporosis respectively. The accompanying bars indicate the 95% CI. Statistically significant OR are accompanied by their *P* values with an asterisk (*).

Analysis revealed significant associations between age (OR 1.08; 95% CI, 1.03-1.12; *P* < .01) and osteoporosis, as well as BMI (OR 0.91; 95% CI, 0.82-0.99; *P* = .04) and vitamin D deficiency score (OR 1.11; 95% CI, 1.00-1.23; *P* = .04) with osteopenia. This indicates an increased risk of osteoporosis with age, and osteopenia with persistent vitamin D deficiency and a reduced risk with a higher BMI (Table 8 and Fig. 4).

**Table 8.** Odds ratios for predictors of osteopenia and osteoporosis risk at baseline

	Odds ratio	Lower CI	Upper CI	*P* value
Normal BMD vs osteopenia				
Intercept	2.37	0.24	23.54	.46
Age	1.01	0.98	1.04	.48
Sex	1.62	0.73	3.61	.24
*BMI*	*0*.*91*	*0*.*82*	*0*.*99*	.04*
Time to symptoms*^a^*	1.00	1.00	1.01	.17
*Vitamin D deficiency score^b^*	*1*.*11*	*1*.*00*	*1*.*23*	.04*
Normal BMD vs osteoporosis				
Intercept	0.36	0.01	16.51	.60
*Age*	*1*.*08*	*1*.*03*	*1*.*12*	*<*.01*
Sex	0.77	0.24	2.48	.66
BMI	0.88	0.76	1.03	.10
Time to symptoms*^a^*	1.00	0.98	1.02	.83
Vitamin D deficiency score*^b^*	1.00	0.87	1.02	.99

Abbreviations: BMD, bone mineral density; BMI, body mass index. Statistically significant odds ratios are in *italics* and their *P* values accompanied by an asterisk (*).

^*a*^Measure for disease severity: time in minutes in direct sunlight triggering symptoms, prior to afamelanotide treatment.

^*b*^Percentage of 25(OH)D measurements below the vitamin D deficiency threshold (< 50 nmol/L) up until baseline.

**Figure 5.**
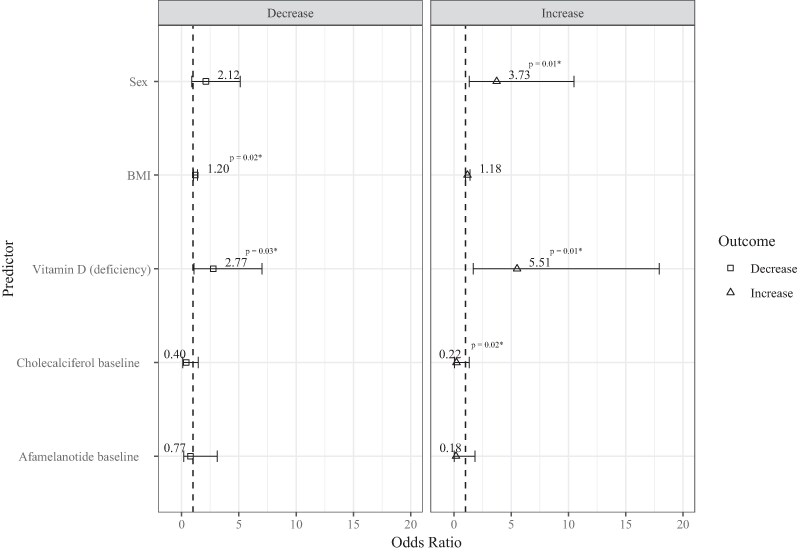
Odds ratios (OR) for predictors of an increase or decrease in BMD. The figure displays the OR along with their corresponding CI, illustrating the associations between the predictors and 2 outcomes: significant increase and significant decrease of the BMD, compared to the reference category of unchanged BMD. The outcome is categorized based on significant changes in BMD compared to baseline, as specified in methods. Those using having both an increase and decrease in BMD and those receiving therapy for osteoporosis, including bisphosphonates and denosumab, were excluded from analysis. The squares and triangles depict the odds ratios for osteopenia and osteoporosis respectively. The accompanying bars indicate the 95% CI. Statistically significant OR are accompanied by their *P* values with an asterisk (*).

When identifying predictors that are associated with change in BMD, we found significant associations between sex (OR 3.73; 95% CI, 1.33-10.49; *P* = .01), baseline vitamin D deficiency (OR 5.51; 95% CI, 1.69-17.92; *P* = .01), cholecalciferol (OR 0.22; 95% CI, 0.04-1.34; *P* = .03) and BMD increase. This indicates higher odds of BMD increase in females compared to males. Furthermore, vitamin D deficiency at baseline leads to higher odds of increasing the BMD at follow-up when compared to having a normal vitamin status. Lastly, the odds of increasing BMD are lower when already taking cholecalciferol (Table 9 and Fig. 5). Afamelanotide, however, did not improve BMD. Additional analysis showed no increase in physical activity or BPAQ scores after initiating afamelanotide treatment (Table 10).

**Table 9.** Odds ratios for predictors of an increase or decrease in BMD

	Odds ratio	Lower CI	Upper CI	*P* value
Unchanged vs increased BMD				
Intercept	0.20	0.00	21.00	.50
*Sex*	*3*.*73*	*1*.*33*	*10*.*49*	.01*
BMI	1.18	1.00	1.40	.05
*Vitamin D (deficiency)^a^*	*5*.*51*	*1*.*69*	*17*.*92*	.01*
*Cholecalciferol baseline*	*0*.*22*	*0*.*04*	*1*.*34*	.02*
Afamelanotide baseline	0.18	0.02	1.84	.15
Unchanged vs decreased BMD				
Intercept	0.03	0.00	1.33	.07
Sex	2.12	0.88	5.12	.10
*BMI*	*1*.*20*	*1*.*03*	*1*.*40*	.02*
*Vitamin D (deficiency)^a^*	*2*.*77*	*1*.*09*	*7*.*01*	.03*
Cholecalciferol baseline*^b^*	0.40	0.11	1.45	.16
Afamelanotide baseline*^b^*	0.77	0.19	3.11	.71

^*a*^Vitamin D (deficiency); categorical variable denoting serum 25-OH vitamin D at baseline, distinguishing between deficiency and sufficiency.

^*b*^Baseline cholecalciferol and afamalenotide use: categorical variable distinguishing between on or off treatment at time of baseline DXA scan. Patients were categorized as on treatment (cholecalciferol or afamelanotide) at baseline if the first prescription was issued before the baseline DXA scan, otherwise, they were categorized as not on treatment.

Abbreviations: BMD, bone mineral density; BMI, body mass index. Statistically significant odds ratios are in *italics* and their *P* values accompanied by an asterisk (*).

**Table 10.** Physical activity according to bone-specific physical activity questionnaire before and after initiating afamelanotide

	Prior to afamelanotide (n = 71)	During afamelanotide (n = 71)	*P* value
BPAQ*^a^*			
Total BPAQ	10.6 (3.8-22.3)	8.3 (1.8-21.3)	.22
Past BPAQ	19.5 (6.1-42.9)	13.5 (1.8-42.4)	.28
Current BPAQ	0.9 (0.4-3.3)	0.7 (0.7-3.0)	.33
Physical activity (times per week)*^a^*	3.0 (1.5-5.0)	3.0 (2.0-6.0)	.72

All data are reported in median (interquartile range). *P* values were calculated with the Wilcoxon signed ranked test.

^*a*^Self-reported. Abbreviation: BPAQ, bone-specific physical activity questionnaire.

Analysis showed that BMI (OR 1.20; 95% CI, 1.03-1.40; *P* = .02) and vitamin D deficiency (OR 2.77; 95% CI, 1.09-7.01; *P* = .03), had a significant associations with a decrease in BMD. Those with a higher BMI had higher odds of decreasing BMD. Moreover, those that had a vitamin D deficiency at baseline had higher odds of decreasing BMD when compared to a normal 25(OH)D status (Table 9 and Fig. 5).

**Figure 6.**
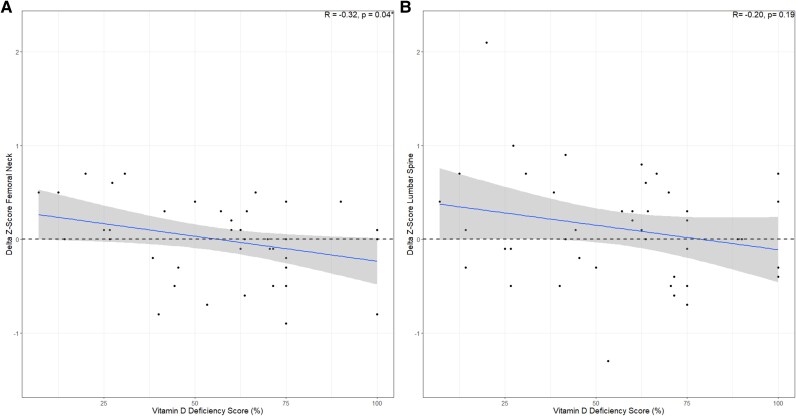
Correlation of Delta Z-scores with vitamin D deficiency in erythropoietic protoporphyria patients with baseline vitamin d deficiency. These figures display the moderate negative association between the delta Z-scores and vitamin D deficiency. The blue solid line represents the regression line. The dashed line indicates the 0 threshold, where values above indicate an increase in Z-scores and those below indicate decrease. The shading indicates the CI. A vitamin D deficiency score of 0% signifies the absence of measured vitamin D deficiency from baseline to follow-up, while a score of 100% indicates persistent vitamin D deficiency measurements. Patients using treatment for osteoporosis, including bisphosphonates or denosumab, were excluded from analysis to assess the true effect of vitamin D. Only patients with a vitamin D deficiency at baseline were included in the analysis. (A) Delta Z-scores of the femoral neck. (B) Delta Z-scores of the lumbar spine. *P* values were calculated using the Spearman Correlation test. Statistically significant *P* values are denoted with an asterisk (*). R, correlation coefficient.

Because vitamin D deficiency at baseline led to both a higher odds for increase and decrease in BMD, we did additional analysis. This analysis looked at the correlation between changes in BMD, expressed in delta Z-scores, and vitamin D scores in those with a vitamin D deficiency at baseline. A moderate negative correlation was found between the vitamin D deficiency score and delta Z-scores for the femoral neck (*r* = −0.32, *P* = .04*) and lumbar spine (*r* = −0.20, *P* = .19) (Fig. 6). This indicates that those with adequate 25(OH)D (> 50 nmol/L) between baseline and follow-up, have BMD improvement, while those with persistent vitamin D deficiency (≤ 50 nmol/L) have a decline in BMD.

## Discussion

In this large single-center longitudinal cohort study we demonstrated that 25(OH)D status plays a crucial role in improving BMD in EPP patients, whereas afamelanotide treatment did not lead to BMD improvement. EPP patients exhibit a higher prevalence of low BMD, with T-scores classified as osteopenia and osteoporosis, compared to the general healthy population. Additionally, there is a higher prevalence of osteoporosis-related fractures. Although vitamin D deficiency is a known risk factor for early-onset osteoporosis, the impact of vitamin D supplementation on BMD in younger populations remains controversial (11-13). This study confirms that cholecalciferol significantly improves BMD in relatively young individuals.

Several predictors were identified as factors associated with improvements in BMD among EPP patients including sex, BMI, 25(OH)D status, and cholecalciferol. Afamelanotide treatment did not have predictive value. Females have a higher likelihood of increased bone density, potentially due to better adherence to cholecalciferol. Previous research indicates that females also show greater improvements in 25(OH)D status compared to males after prescribing cholecalciferol treatment (25).

EPP patients already taking cholecalciferol at baseline are less likely to improve their BMD, while individuals with baseline vitamin D deficiency have a higher chance of both increasing and decreasing in their BMD. These findings suggest that BMD improvement depends on whether the deficiency is adequately addressed. Failure to address vitamin D deficiency may result in further deterioration of BMD, whereas adequate supplementation may lead to improved 25(OH)D levels and subsequently a higher chance of increasing BMD. Additional correlation analysis reinforces this observation, indicating that individuals with baseline vitamin D deficiency who maintain continuous sufficient 25(OH)D levels show an improvement in BMD, whereas those with measured deficiency show a decline.

Elevated BMI increases the odds of further deterioration of the BMD. This is an unexpected finding, seeing that increased weight has an osteogenic effect and is therefore generally considered protective (28). One possible explanation could be that overweight patients have been shown to have lower 25(OH)D levels (29). This could be due to even less sun exposure or the storage of 25(OH)D in adipose tissue, which leads to reduced availability in the bloodstream (30). Furthermore, obesity and overweight decreases the effect of vitamin D supplementation in adults (31). Lastly, obese individuals usually participate in less physical activity (32), which could lead to further deterioration of the BMD.

Contrary to our hypothesis, afamelanotide treatment was not associated with an increase in BMD. While it is known to enhance light tolerance (8, 9), it appears insufficient to stimulate dermal synthesis of vitamin D (25). Additionally, patients did not report an increased amount of physical activity after initiating afamelanotide treatment.

EPP patients have a higher odds of developing osteoporosis as they age, while a high BMI is protective for developing osteopenia. This aligns with the risk factors in the general population (33). The risk of developing osteopenia is also higher in patients with persistent vitamin D deficiency, which is a known cause for early-onset osteoporosis (2, 17). Sex and disease severity measured in time to symptoms, do not have additional predictive value.

Our study confirmed that when osteoporosis is diagnosed based on T-scores from DXA scans, the prevalence of osteopenia and osteoporosis is strikingly high among EPP patients and manifests at a relatively young age. When comparing them to the healthy population of the same age, sex, and ethnicity using Z-scores, 82.7% of the EPP patients exhibited a baseline Z-score below 0 SD. While the prevalence of early-onset osteoporosis in the general population is unknown (2), by definition, only 0.6% of the healthy population aged 20 to 40 years should suffer from osteoporosis and only 15.9% should have osteopenia (17). In contrast, among EPP patients, these rates are substantially higher, with 10.7% affected by osteoporosis and 51.8% by osteopenia. Among individuals aged 50 years and older, the prevalence of osteoporosis in EPP patients was much higher at 33.3% compared with 6.3% to 14.3% in the general European population (34). Interestingly, within the EPP population, an equal number of men and women are affected by osteoporosis, in contrast to the general population where women are predominantly impacted (34).

This is likely explained by the fact that in EPP patients, low BMD is most probably not due to true osteoporosis, which results from the loss of bone matrix, but rather from the demineralization of the osteoid, a condition known as osteomalacia (35). This distinction clarifies why cholecalciferol supplementation improves BMD in these patients by promoting proper mineralization, and why those already taking cholecalciferol at baseline are less likely to improve. Although we did not specifically investigate osteomalacia-related symptoms, our clinical experience is that EPP patients did not have clear clinical signs of osteomalacia, such as musculoskeletal pain and weakness (36). However, given the nonspecific nature of these symptoms, it is possible subtle manifestations were overlooked. No physical examinations, such as applying pressure on the sternum, tibia, or radius to elicit discomfort or pain, were conducted to assess bone pain, a diagnostic indicator of osteomalacia (36). Bone biopsies were not performed for its definite diagnosis. In this manuscript, the terms *osteoporosis* and *osteopenia* refer to the low BMD observed on DXA scans.

The lifetime risk of any osteoporotic fracture in the general healthy population is substantial, ranging from 40% to 50% for women and 13% to 22% for men (20). We observe an even higher prevalence in this EPP cohort, with 66.7% of the women and 30.8% of the men having experienced fractures at an osteoporosis-related site, even though our cohort is relatively young. However, there is no evidence that these fractures were directly caused by osteoporosis. Furthermore, it is important to note that in osteomalacia, preferred fracture sites include the metatarsals, which are now classified as non-osteoporosis-related (35, 37).

Bisphosphonates are an effective treatment option in EPP patients with osteoporosis aged 45 and older. However, the risk of rare but severe side-effects, including adverse effects in pregnancies in women of childbearing age (38), aseptic bone necrosis of the jaw, and atypical spontaneous femur fractures should be taken into consideration when prescribing this medication (2, 18). Previous literature has shown that bisphosphonates lead to a BMD increase, in patients with early-onset osteoporosis, although evidence on fracture reduction is lacking (2). Therefore, especially in young patients, adequately addressing vitamin D deficiency and providing lifestyle recommendations on alcohol consumption, smoking cessation, and physical activity should be the initial approach. The use of bisphosphonates in young patients should be limited to those who have fragility fractures (2) or additional risk factors, according to international guidelines (18).

Strengths of this study include a large cohort for EPP, a rare disease, along with longitudinal data, and some prospectively collected predictors. Limitations arise from small sample sizes in certain stratified groups, such as osteoporosis and increased BMD group, limiting prediction modeling. Missing values on smoking, alcohol intake, and physical activity limited possibilities for including them as predictors in our model. Additionally, the vitamin D deficiency score did not consider the duration or depth of vitamin D deficiency. Furthermore, most 25(OH)D measurements were taken during spring, summer, and autumn, while winter has been shown to have lower values, even in EPP patients (9, 25). Therefore, it is likely that the vitamin D deficiency score used is an overestimation. Accounting for duration, and severity of 25(OH)D deficiency, and seasonal variation of 25(OH)D could lead to stronger associations between vitamin D status and BMD increase. Despite these limitations, the study provides valuable insights into real-world circumstances.

In conclusion, our findings establish the high prevalence of osteoporosis and fractures in EPP at relatively young age, emphasizing the importance of adequately addressing vitamin D deficiency for both osteoporosis prevention and BMD improvement. Afamelanotide did not improve BMD. We suggest that future guidelines on the treatment of EPP include continuous 25(OH)D monitoring, supplementation, and a one-time DXA scan for all adult EPP patients. Follow-up DXA scans can be considered to assess treatment effects or BMD deterioration in patients ineffectively addressing their vitamin D deficiency. This population serves as a natural model for lack of sunlight exposure and vitamin D deficiency, underlining the importance of lifelong adequate 25(OH)D levels for bone health.

## Data Availability

Datasets generated during and/or analyzed during the current study are not publicly available but are available from the corresponding author on reasonable request.

## Abbreviations

25(OH)D: 25-hydroxyvitamin D
BMD: bone mineral density
BPAQ: bone-specific physical activity questionnaire
DXA: dual-energy x-ray absorptiometry
EPP: erythropoietic protoporphyria
IQR: interquartile range
WHO: World Health Organization
